# Prognostic Utility of Neutrophil-to-Lymphocyte Ratio on Adverse Clinical Outcomes in Patients with Severe Calcific Aortic Stenosis

**DOI:** 10.1371/journal.pone.0161530

**Published:** 2016-08-22

**Authors:** Kyoung Im Cho, Sang Hoon Cho, Ae-Young Her, Gillian Balbir Singh, Eun-Seok Shin

**Affiliations:** 1 Department of Cardiology, Kosin University School of Medicine, Busan, South Korea; 2 Department of Statistics and Actuarial Science, Soongsil University, Seoul, Korea; 3 Division of Cardiology, Department of Internal Medicine, Kangwon National University School of Medicine, Chuncheon, South Korea; 4 Department of Cardiology, Ulsan University Hospital, University of Ulsan College of Medicine, Ulsan, South Korea; Osaka University Graduate School of Medicine, JAPAN

## Abstract

**Background:**

Inflammation is an important factor in the pathogenesis of calcific aortic stenosis (AS). We aimed to evaluate the association between an inflammatory marker, neutrophil-to-lymphocyte ratio (NLR) and major adverse cardiovascular events (MACE) in patients with severe calcific AS.

**Methods:**

A total of 336 patients with isolated severe calcific AS newly diagnosed between 2010 and 2015 were enrolled in this study. Using Cox proportional hazards (PH) regression models, we investigated the prognostic value of NLR adjusted for baseline covariates including logistic European System for Cardiac Operative Risk Evaluation score (EuroSCORE-I) and undergoing aortic valve replacement (AVR). We also evaluated the clinical relevance of NLR risk groups (divided into low, intermediate, high risk) as categorized by NLR cutoff values. MACE was defined as a composite of all-cause mortality, cardiac death and non-fatal myocardial infarction during the follow-up period.

**Results:**

The inflammatory marker NLR was an independent prognostic factor most significantly associated with MACE [hazard ratio (HR), 1.06; 95% confidence interval (CI), 1.04–1.09; p-value <0.001]. The goodness-of-fit and discriminability of the model including EuroSCORE-I and AVR (loglikelihood difference, 15.49; p-value <0.001; c-index difference, 0.035; p-value = 0.03) were significantly improved when NLR was incorporated into the model. The estimated Kaplan-Meier survival rates at 5 years for the NLR risk groups were 84.6% for the low risk group (NLR ≤ 2), 67.7% for the intermediate risk group (2 < NLR ≤ 9), and 42.6% for the high risk group (NLR > 9), respectively.

**Conclusion:**

The findings of the present study demonstrate the potential utility of NLR in risk stratification of patients with severe calcific AS.

## Introduction

Calcific aortic stenosis (AS) represents a major public health burden associated with progressively increasing morbidity and mortality [1, 2]. This is especially so with a rapidly aging global society and the relatively high prevalence of AS being observed among the elderly. A novel approach enabling the risk stratification of AS patients would undoubtedly furnish an invaluable tool to greatly facilitate clinical decisions. Recent endeavors towards researching the complex abnormalities in risk stratification of AS patients have identified several potential biomarkers. Overall however, there remains a lack of biomarkers to complement the prognostic use of simple risk scores, although levels of natriuretic peptides [3] and high-sensitivity troponin may have a predictive role in AS.

There is an association between inflammation and remodeling of calcific aortic valve disease where inflammation and calcification are believed to play key roles in the disease [4]. Although high-sensitivity C-reactive protein (hs-CRP), a marker of systemic inflammation, may have a role in identifying patients in the early stages of calcific AS [5], there are conflicting study results with respect to the association between hs-CRP level and prognosis of calcific AS [6, 7]. Currently, neutrophil-to-lymphocyte ratio (NLR), a simple and inexpensive method for assessing inflammation, is being investigated as a new predictor of cardiovascular risk as an important inflammatory marker [8, 9]. Recent meta-analysis indicates that NLR is a predictor of all-cause mortality and cardiovascular events [10]. Furthermore, NLR has been identified as an independent factor associated with coronary calcium score [11]. The objective of this study was to investigate the prognostic value of NLR as an independent predictor for adverse clinical outcomes in patients with severe calcific AS and additionally evaluate the clinical relevance of NLR in stratifying AS patients into heterogeneous risk groups.

## Methods

### Study population

A total of 336 symptomatic or asymptomatic patients newly diagnosed with isolated severe calcific AS between January 2010 and January 2015 were retrospectively enrolled in this study from two university hospitals in South Korea. The definition of severe calcified AS was based on the American Society of Echocardiography (ASE) guidelines (peak velocity ≥4 m/s or mean pressure gradient (PG) > 40 mm Hg in the presence of normal left ventricular (LV) function or calculated aortic valve area (AVA) <1.0 cm^2^) [12]. In cases where LV systolic dysfunction co-existed with mean PG 30–40 mmHg and AVA of 1.0 cm^2^ (low-flow low-gradient AS), dobutamine stress echocardiography was used to discriminate severe calcific AS causing LV systolic dysfunction (n = 25). Patients with systemic diseases and those on treatment with agents affecting white blood cell count including patients with hematological disorders, malignancies, chemotherapy treatment, evidence of concomitant inflammatory disease, acute infection, chronic inflammatory conditions, history of corticosteroid therapy in the preceding 3 months, prior valve replacement surgery, secondary hypertension or end-stage renal disease on dialysis were excluded from this study. In addition, patients with valvular disease in valves other than aortic valve (AV) such as bicuspid AV, rheumatic AS or severe regurgitation of valves were excluded. Clinical risk factors (age, sex, diabetes mellitus, hypertension, hyperlipidemia, smoking status) and New York Heart Association (NYHA) functional class were analyzed by retrospective chart review. European System for Cardiac Operative Risk Evaluation score (EuroSCORE-I) was calculated in each patient via available online tools (http://www.euroscore.org) in order to reflect coexisting clinical risk [13]. This study complies with the Declaration of Helsinki and was approved by the Ulsan University Hospital Institutional Review Board with written informed consent obtained from all participants.

### Laboratory analysis

Complete blood counts, which included total white blood cells, neutrophils, lymphocytes, and platelets as well as echocardiography were obtained at the time of admission as part of routine clinical work-up. Liver enzymes, glucose, creatinine, lipid profiles, N-terminal proBNP (NT- proBNP), and hs-CRP were also measured in all patients. NT-proBNP was measured using immunoradiometric assays with a commercial kit for BNP (COBAS 6000 E601 module, Roche Diagnostics HITACH HIGH TECH CORP., Indianapolis, IN, USA), and hs-CRP was measured using fully automated turbid immunometry (Advia 1800, Siemens). NLR was calculated as the ratio of neutrophil count to lymphocyte count.

### Echocardiography

Echocardiographic assessments were performed using a Sonos 5500 system (Philips Medical Systems, Bothell, WA) with standardized imaging techniques. A comprehensive echocardiographic examination including M-mode echocardiography, two-dimensional echocardiography, and conventional and color Doppler ultrasonography were performed according to European Society of Echocardiography and ASE criteria [12]. The peak velocity across the valve was measured with continuous-wave Doppler from the window with the strongest velocity signal. AVA was calculated using the continuity equation, and ejection fraction (EF) was calculated using the biplane Simpson method. Measurements of thicknesses of interventricular septum and posterior wall, diameter of the LV cavity, and LV mass index (LVMI) were calculated from the ratio between LV mass. Body mass index (BMI) was also calculated.

### Clinical outcomes and definitions

The endpoint of the study was major adverse cardiovascular events (MACE) in the follow-up period defined as a composite of all-cause mortality, cardiac death and non-fatal MI. Intra-operative deaths as well as deaths during the follow-up periods were included in the analysis. Deaths were classified as cardiac or non-cardiac following review of medical records, including autopsy records and death certificates, which were available for all cases. Nonfatal MI was defined using the definitions of the European Society of Cardiology, American College of Cardiology, American Heart Association, and World Heart Federation [14].

### Statistical analysis

Differences in baseline characteristics between patient groups (with MACE and without MACE) were tested using t-statistics for continuous variables. In the case of categorical variables, we applied the chi-square test if expected frequencies equal at least 5; otherwise, the Fisher exact test was utilized.

Cox proportional hazards (PH) regression models were employed to investigate the prognostic value of NLR adjusted by the baseline covariates including EuroSCORE-I, AVR, and NLR. The independent prognostic factors significantly associated with MACE were selected in stepwise fashion avoiding over-parameterization. The underlying proportional hazards assumptions of the Cox PH models were verified by Schoenfeld residual tests. The goodness-of-fit and discriminability of Cox PH models were assessed by likelihood ratio tests and Harrell’s c-index, respectively [15]. The confidence intervals of the C-index were constructed by the nonparametric bootstrap method [16].

Upon the verification of the prognostic value of NLR as a continuous variable, we further investigated the clinical relevance of the categorized NLR that assign patients into three risk groups (low, intermediate, high). The cutoff values for the NLR risk groups were carefully determined to minimize information loss in terms of loglikelihood difference as discretizing the continuous variable of NLR [17].

The Kaplan-Meier method was applied to estimate survival curves for categorized NLR risk groups without imposing any parametric assumption. In hypothesis testing, a significance level of 0.05 was chosen. All statistical analyses were performed using R (http://www.r-project.org).

## Results

### Characteristics of the study population

**Fig 1:**
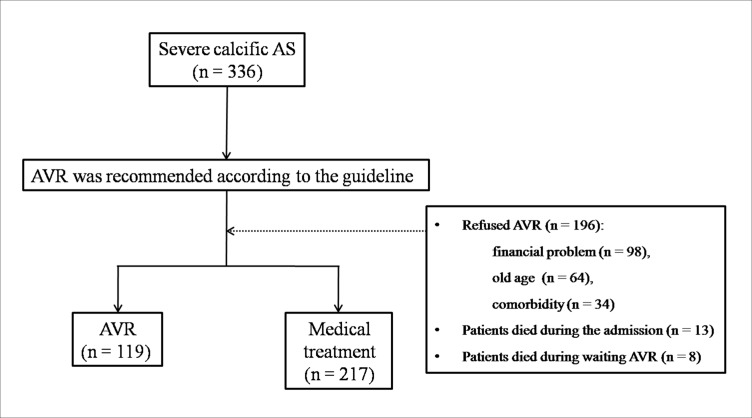
Clinical layout of severe calcific aortic stenosis (AS) cohort. Although aortic valve replacement (AVR) was recommended according to the guideline, only 119 patients underwent AVR because of various situations.

Among the 336 patients enrolled in the study population, 166 (49.4%) were male and the mean age of the patients was 70.1 ± 12.0 years. Fig 1 showed the detail information for the presentation of the cohort.

The median follow-up time was estimated to be 33 months using the reverse Kaplan-Meier method [18]. The clinical characteristics of the study population according to the status of MACE are summarized in Table 1.

**Table 1:** Baseline Clinical Characteristics According to Major Cardiovascular Event.

	MACE (+) (n = 82)	MACE (-) (n = 254)	*p*-value
**Age, years**	72.3 ± 10.4	69.4 ± 12.4	0.014
**BMI, kg/m**^**2**^	22.7 ± 3.10	23.6 ± 3.10	0.034
**Male, n (%)**	38 (46.3%)	128 (50.4%)	0.609
**Systolic BP, mmHg**	126.5 ± 24.1	126.9 ± 20.1	0.909
**Diastolic BP, mmHg**	72.0 ± 15.4	71.4 ± 12.6	0.806
**Current smoker, n (%)**	14 (17.1%)	35 (13.8%)	0.579
**Hypertension, n (%)**	36 (43.9%)	119 (46.9%)	0.735
**Diabetes mellitus, n (%)**	17 (20.7%)	59 (23.2%)	0.750
**Dyslipidemia, n (%)**	17 (20.7%)	74 (29.1%)	0.178
**Previous CVA, n (%)**	9 (11.0%)	28 (11.0%)	1.000
**Significant CAD, n (%)**	18 (22.0%)	37 (14.6%)	0.162
**Previous PCI**	14 (17.1%)	18 (7.1%)	0.014
**EuroSCORE-I**	8.5 ± 9.4	6.0 ± 5.8	0.024
**NYHA functional class, n (%)**			0.162
**Class I**	22 (26.8%)	83 (34.0%)	
**Class II**	26 (31.7%)	87 (35.7%)	
**Class III**	21 (25.6%)	54 (22.1%)	
**Class IV**	13 (15.9%)	20 (8.2%)	
**Aortic valve replacement, n (%)**	21 (25.6%)	98 (38.6%)	0.045

Values are means ± SDs for continuous variables or frequencies (percentages) for categorical variables.

BMI, body mass index; BP, blood pressure; CAD, coronary artery disease; CVA, cerebrovascular accident; MACE, major adverse cardiovascular event; NYHA, New York Heart Association; PCI, percutaneous coronary intervention

Compared to the patient group with no MACE occurrence (MACE (-)), the patient group with MACE (MACE (+)) consisted of older patients, had lower BMI, higher heart rate and higher EuroSCORE-I, and underwent more frequent percutaneous coronary interventions and less frequent AVR (all p-values <0.05). In Table 2, the laboratory characteristics of the study population are compared between the two patient groups. Compared to MACE (-), MACE (+) showed reduced lymphocyte count and hematocrit, higher platelet density width and serum creatinine, and correspondingly lower estimated glomerular filtration rate (eGFR) (all p-values <0.05). S1 Fig showed the empirical joint and marginal distributions of lymphocyte and neutrophil depicted by a scatter plot and histograms.

**Table 2:** Baseline Laboratory Characteristics According to Major Cardiovascular Event.

	MACE (+) (n = 82)	MACE (-) (n = 254)	*p*-value
**White blood cell, x10**^**9**^**/L**	8.8 ± 4.1	7.8 ± 3.5	0.049
**Neutrophil, %**	69.1 ± 15.0	66.1 ± 37.3	0.293
**Lymphocyte,%**	20.2 ± 11.6	25.5 ± 11.4	<0.001
**Monocyte, %**	6.5 ± 2.9	7.3 ± 3.0	0.035
**Hemoglobin, g/dL**	12.0 ± 4.1	12.2 ± 2.0	0.724
**Hematocrit, %**	34.7 ± 6.0	36.1 ± 5.7	0.070
**Red cell distribution width**	14.9 ± 2.2	14.4 ± 2.4	0.062
**Platelets, x10**^**9**^**/L**	220.1 ± 88.9	200.5 ± 77.4	0.076
**NLR**	7.1 ± 10.0	4.1 ± 4.7	0.009
**hs-CRP, mg/L**	4.4 ± 6.7	2.1 ± 4.0	0.034
**Serum creatinine, mg/dL**	1.7 ± 2.4	1.3 ± 1.4	0.182
**eGFR, ml/min/1.73m**^**2**^	62.0 ± 34.2	71.3 ± 29.4	0.040
**Total cholesterol, mg/dL**	167.1 ± 42.1	169.5 ± 42.3	0.648
**LDL cholesterol, mg/dL**	94.4 ± 32.8	100.0 ± 39.0	0.286
**HDL cholesterol, mg/dL**	43.3 ± 14.6	45.0 ± 14.3	0.399
**Triglycerides, mg/dl**	101.0 ± 89.8	109.9 ± 69.8	0.471
**NT-proBNP, ng/mL**	7058.0 ± 8026.2	3359.1 ± 6079.8	0.006

Values are means ± SDs for continuous variables or frequencies (percentages) for categorical variables.

NLR, neutrophil-to-lymphocyte ratio; hs-CRP, high sensitivity C-reactive protein; eGFR, estimated glomerular filtration rate according to the Modification of Diet in Renal Disease equation; LDL, low density lipoprotein;HDL, high density lipoprotein; NT-proBNP, n-terminal pro brain natriuretic peptide.

**Fig 2:**
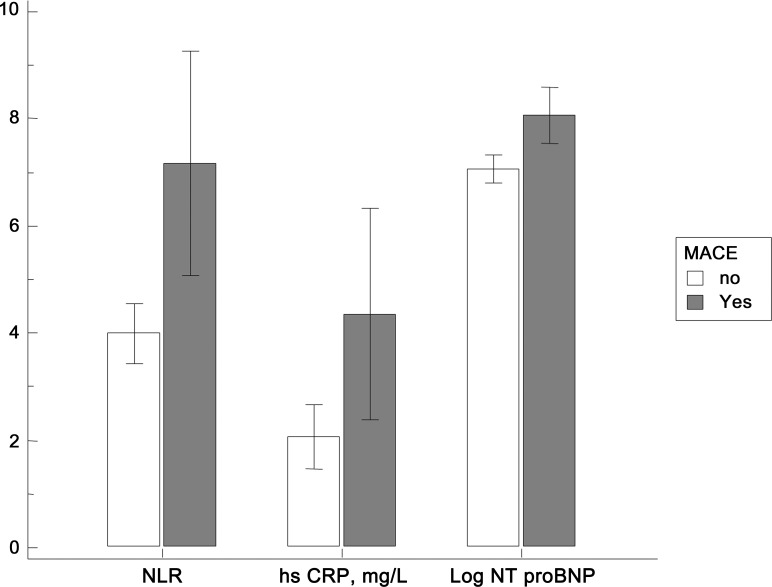
Neutrophil-to lymphocyte ratio (NLR), N-terminal pro-brain natriuretic peptide (NT-proBNP), and high-sensitivity C-reactive protein (hs-CRP) according to major adverse cardiovascular events (MACEs) and New York Heart Association (NYHA) functional class.

As depicted in Fig 2, significantly higher NLR, NT-proBNP, and hs-CRP values were also observed in MACE (+).

According to the echocardiographic characteristics of the study population listed in Table 3, MACE (+) had reduced AVA and LVEF, increased trans-valvular PG and left atrial diameter, and higher ratio of peak earlier mitral filling velocity to mitral annulus velocity (E/Ea) compared to MACE (-) (all p-values <0.05).

**Table 3:** Baseline Echocardiographic Characteristics According to Major Cardiovascular Event (MACE).

	MACE (+) (n = 82)	MACE (-) (n = 254)	*p*-value
**AV area, cm**^**2**^	0.7 ± 0.2	0.8 ± 0.2	0.018
**AV maximal velocity, cm/s**	4.5 ± 0.8	4.3 ± 0.8	0.011
**AV maximal pressure gradient, mmHg**	84.2 ± 29.3	75.7 ± 27.8	0.023
**AV mean pressure gradient, mmHg**	51.4 ± 19.2	44.1 ± 18.1	0.003
**LVEDD, mm**	47.7 ± 8.0	46.9 ± 8.0	0.474
**LVEDV, ml**	93.3 ± 39.3	90.6 ± 42.0	0.597
**LVESD, mm**	33.4 ± 9.4	30.7 ± 8.6	0.027
**LVESV, ml**	43.2 ± 29.9	36.0 ± 28.8	0.063
**LV ejection fraction, %**	57.2 ± 15.0	63.0 ± 12.9	0.002
**LV mass index, kg/m**^**2**^	160.9 ± 44.9	156.8 ± 58.2	0.540
**IVSd, mm**	13.5 ± 2.9	14.2 ± 3.6	0.073
**PWTd, mm**	12.6± 2.2	12.4 ± 2.7	0.622
**RWT**	0.5± 0.1	0.5± 0.2	0.875
**Left atrial diameter, mm**	44.7± 8.5	42.5± 8.8	0.052
**E/Ea**	22.5 ± 10.6	18.9 ± 8.9	0.018

Values are means ± SDs for continuous variables or frequencies (percentages) for categorical variables.

AV, aortic valve; LV, left ventricular; LVEDD, left ventricular end-diastolic diameter; LVEDV, left ventricular end-diastolic volume; LVESD, left ventricular end-systolic diameter; LVESV, left ventricular end-systolic volume; IVSd, diastolic interventricular septal wall thickness; PWTd, diastolic posterior wall thickness; RWT, relative wall thickness; E, peak early diastolic mitral filling velocity; Ea, mitral annular velocity; EF, ejection fraction.

### Prognostic value of NLR in patients with severe calcific AS

According to the Cox PH regression analysis, high EuroSCORE-I and a history of AVR were significantly associated with MACE. Also, the inflammatory variable, NLR, was the independent prognostic factor most significantly associated with MACE (hazard ratio (HR), 1.06; 95% confidence interval (CI), 1.04–1.09; p-value < 0.001; Table 4). S2 Fig showed the empirical distributions of the continuous type of variables present in Table 4 utilizing box-plots

**Table 4:** Cox Proportional Hazards Regression Analysis Regarding Major Cardiovascular Event.

	Simple			Multivariable		
	HR	95% CI	*p*-value	HR	95% CI	*p*-value
**Age, years**	1.03	1.01 to 1.05	0.004			
**BMI, kg/m**^**2**^	0.92	0.86 to 0.99	0.032			
**Heart rate, min/sec**	1.01	1.00 to 1.02	0.006			
**EuroSCORE-I**	1.07	1.05 to 1.10	<0.001	1.05	1.03 to 1.08	<0.001
**PCI, n (%)**	2.49	1.39 to 4.45	0.002			
**Aortic valve replacement, n (%)**	0.43	0.26 to 0.70	0.001	0.52	0.31 to 0.87	0.012
**Lymphocyte,%**	0.95	0.93 to 0.97	<0.001			
**Hematocrit, %**	0.95	0.91 to 0.98	0.004			
**Red cell distribution width**	1.10	1.02 to 1.17	0.009			
**NLR**	1.07	1.05 to 1.09	<0.001	1.06	1.04 to 1.09	<0.001
**hs-CRP, mg/L**	1.09	1.04 to 1.14	<0.001			
**eGFR, ml/min/1.73m**^**2**^	0.99	0.98 to 1.00	0.046			
**NT-proBNP, ng/mL**	1.00	1.00 to 1.00	<0.001			
**LVESD, mm**	1.02	1.00 to 1.05	0.043			
**LV ejection fraction, %**	0.98	0.97 to 1.00	0.013			
**E/Ea**	1.04	1.01 to 1.04	0.002			

MACE, major adverse cardiovascular event; HR, hazard ratio; CI, confidence interval; BMI, body mass index; PCI, percutaneous coronary intervention; NLR, neutrophil to lymphocyte ratio; hs-CRP, high sensitivity C-reactive protein; eGFR; estimated glomerular filtration rate; NT-proBNP; n-terminal pro brain natriuretic peptide; LVESD, left ventricular end-systolic diameter; LV, left ventricular. E, peak early diastolic mitral filling velocity; Ea, mitral annular velocity.

The likelihood and C-index estimates of Cox PH regression models are summarized in Table 5. The inflammatory variable, NLR, has the highest likelihood value and C-index among three variables including AVR, EuroSCORE-I, and NLR. The incremental effect of NLR on MACE was also demonstrated by comparing three different Cox PH regression models: Model 1 includes AVR; Model 2 includes AVR and EuroSCORE-I; Model 3 includes AVR, EuroSCORE-I, and NLR, respectively. According to the comparison between Model 2 and Model 3, NLR significantly improved the goodness-of-fit and discriminability of the Model 2 including AVR and EuroSCORE-I (loglikelihood difference, 15.49; p-value < 0.001; c-index difference, 0.035; p-value = 0.03).

**Table 5:** Comparison of the Goodness-of-fit and discriminability of Cox Proportional Hazards Regression Models.

	Goodness-of-fit			Discriminability			
	Loglik	Diff.	*p*-value*	C-index	Diff.	95% CI^+^	*p*-value^+^
**Model 1**	-397.69			0.58			
**Model 2**	-389.08			0.66			
**Model 3**	-381.49			0.70			
**Model 1 vs. Model 2**		8.61	<0.001		0.079	0.038 to 0.118	0.002
**Model 1 vs. Model 3**		16.35	<0.001		0.114	0.068 to 0.164	<0.001
**Model 2 vs. Model 3**		7.74	<0.001		0.035	0.002 to 0.070	0.033

Model 1, AVR; Model 2, AVR + EuroSCORE-I; Model 3, AVR + EuroSCORE-I + NLR; Loglik, loglikelihood; Diff., difference

C-index, Harrell’s concordance index; *p*-value* is based on the loglikelihood ratio test

95% confidential interval (CI)^+^ and *p*-value^+^ are based on the nonparametric bootstrap method.

The 95% CI and p-value of the difference of the C-indexes were calculated using the empirical distribution of the indexes based on 2,000 bootstrap re-samples. When we extended the definition of MACE including a composite of all-cause mortality, cardiac death, non-fatal MI and heart failure admission, NLR still remains as the independent prognostic factor most significantly associated with MACE after properly adjusting the effect of AVR (S1 Table). S3 Fig showed the empirical distributions of NLR according to sub-patient groups classified by the status of AVR (with or without AVR) and by the status of MACE (with or without MACE).

### Optimized cutoff values of NLR

The upper and lower cutoff values of NLR were optimized to minimize the information loss in categorizing NLR and were estimated to be 2 and 9, respectively. Among a total of 336 patients, 112 (33.3%), 186 (55.4%), and 38 (11.3%) patients were assigned into three risk groups as follow: the low risk group if NLR ≤2, the intermediate risk group if 2 <NLR ≤9, and the high risk group if NLR >9. As shown in Table 6, the categorized NLR was also a significant independent prognostic factor. Compared with the low risk group, the adjusted HR of the intermediate risk group and the high risk group were estimated to be 1.90 (95% CI, 1.07–3.38; p-value = 0.027) and 4.85 (95% CI, 2.38–9.90; p-value <0.001), respectively.

**Table 6:** Effect of neutrophil to lymphocyte ratio (NLR) Risk Groups on Major Adverse Cardiovascular Event.

	No. of patients (%)	HR*	95% CI	*p*-value
**Low (NLR ≤2)**	112 (33.3%)	Reference		
**Intermediate (2< NLR ≤9)**	186 (55.4%)	1.90	1.07 to 3.38	0.027
**High (NLR >9)**	38 (11.3%)	4.85	2.38 to 9.90	<0.001

HR*, hazard ratios adjusted by covariates including Logistic EuroSCORE I and Aortic valve replacement (AVR) using a Cox proportional hazards regression model.

### Clinical value of the NLR risk classification

**Fig 3:**
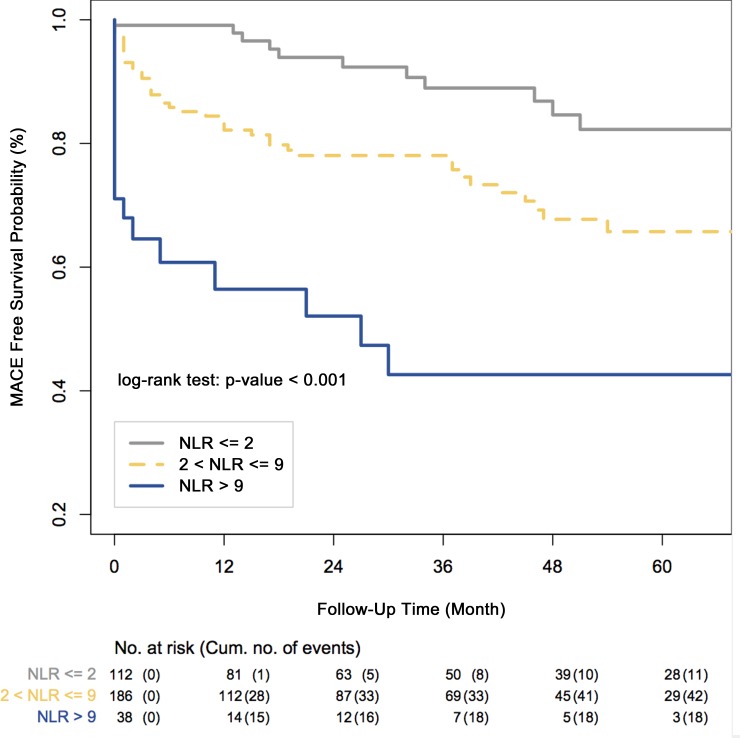
Major Adverse Cardiovascular Event free survival curves of neutrophil to lymphocyte ratio (NLR) risk groups estimated by the Kaplan-Meier method.

As depicted in Fig 3, there exists a statistically significant separation among the estimated Kaplan-Meier survival curves of the NLR risk groups (p-value <0.001).

The estimated MACE free survival rates at 5 years for the NLR risk groups were 84.6% for the low risk group, 67.7% for the intermediate risk group, and 42.6% for the high risk group, respectively (Table 7).

**Table 7:** Kaplan Meier Survival Estimates at 5 Years for NLR Risk Groups.

Group	No. of patients	5-year survival rate (%)	95% CI
**All patients**	336	70.3	64.2 to 77.1
**Low (NLR ≤2)**	112	84.6	75.9 to 94.3
**Intermediate (2< NLR ≤9)**	186	67.7	59.4 to 77.3
**High (NLR >9)**	38	42.6	27.4 to 66.4

NLR, neutrophil to lymphocyte ratio.

## Discussion

In the present study of 336 consecutive patients diagnosed with severe calcific AS for a given period, we observed the natural history of the study patients and all suitable patients underwent AVR. The unique finding of this study was that NLR, a new predictive inflammatory marker, in cardiovascular disease was an important independent predictor of MACE in patients with severe calcific AS. In addition, the incorporation of NLR into a model with EuroSCORE-I, a validated clinical risk score for patients with calcific AS [16, 19], and AVR, a procedural factor significantly improved the goodness-of-fit and the discriminability for the long-term clinical outcomes in patients with severe calcific AS. This is the first study, to our knowledge, to explore adverse outcomes in patients with severe calcific AS via the combination of the inflammatory marker and the clinical risk score.

Predicting outcomes in patients recently diagnosed with calcific AS is clinically relevant, and the assessment of hemodynamic obstruction defined by echocardiographic indexes including trans-valvular PG and AVA is suboptimal because of technical difficulties and poor association with symptoms. Apart from natriuretic peptides, biomarkers have not played a significant role in the evaluation or management of patients with calcific AS [3], although their role in clinical management is not clearly defined. Given the association between inflammation and calcific AS including calcification, fibrosis, and lipid storage [1, 20], which indicate the morphological changes seen in atherosclerosis, validation of inflammatory markers in coronary artery disease and calcific AS might be valuable. In this regard, hs-CRP, a useful predictive biomarker of systemic inflammation and coronary atherosclerosis [21], has also been investigated to assess its relationship with the severity, progression rate and prognosis of calcific AS. Although hs-CRP might have a role in identifying patients in the early stages of calcific AS [5], no relationship has been found in a large population-based cohort [22], with conflicting results noted for the association between hs-CRP level and prognosis of calcific AS [6, 7].

Recently, NLR was identified as an important inflammatory marker and is an inexpensive, routine, reproducible and widely available test. Although NLR is known as potential predictor of cardiovascular risk in patients undergoing percutaneous coronary intervention [9, 23, 24], the relationship between NLR and calcific AS has not been sufficiently investigated. However, there Although EuroSCORE-I score is a validated operative risk score for patients with AS [16, 19], it is calculated using age, NYHA functional class, LV systolic function, and renal function, all of which were associated with the long-term likelihood of MACE in patients with severe calcific AS. In the present study, high EuroSCORE-I was significantly associated with MACE. The inflammatory variable, NLR, was the independent prognostic factor most significantly associated with MACE. This corresponds with the result by Avci et al., who showed that an increased NLR is related to the severity of calcific AS and LV systolic dysfunction in patients with severe calcific AS [25]. As expected, the procedural factor AVR was also important for the long-term prognosis, and because 119 patients had AVR during follow-up, we utilized the multivariable Cox hazard regression model for adjusting the heterogeneous effects of AVR. Interestingly, the incorporation of NLR into a model with AVR and EuroSCORE-I significantly improved the discriminability of the model measured by Harrel’s concordance index. Our observation of an effective method for stratifying mortality risk beyond the EuroSCORE-I score based on inflammatory marker such as NLR in combination with more accurate clinical risk scores might be useful in identifying subgroups of patients likely to exhibit a dismal prognosis even with valve replacement. We believe that it would be certainly an interesting topic for a future study investigating whether or not it is possible to predict the severity of AS based on the level of NLR. This future study would be plausible if there exist a wide spectrum of patients in the study population in terms of the severity of AS. However, our study population includes patients only with isolated severe calcific AS and thus we may pursue the investigation in the future when we obtain appropriate data sets.

Several limitations should be considered when interpreting the findings of the present study. While the study cohort consisted exclusively of severe calcific AS patients diagnosed from echocardiography, the initial presenting symptoms were heterogeneous. Secondly, despite 70% of the patients in our cohort experiencing heart failure symptoms, only 35% of patients underwent AVR because they were either high risk or refused to undergo surgical AVR. As there are no observational studies comparing AVR and medical observation after diagnosis of calcific AS in Korea, we cannot assume that an accurate proportion of severe calcific AS patients underwent AVR. However, compared to Western countries, our results might reflect the Asian cultural trend of refusing surgery in old age as well as the poor accessibility of transcatheter AVR (TAVR) in Korea due to its high cost. Indeed, as TAVR is not widely performed in Korea, our patients at higher risk were not referred for consideration of TAVR, which limits the generalization of our results. However, considering other studies showing that medical therapy tends to be chosen for older patients with comorbidities [26] and the performance of AVR in 42% of patients with low-gradient severe AS during a mean follow-up of 46 months [27], our result might reflect the real practice in the treatment of severe calcific AS in Korea. Additionally, we used the EuroSCORE-I score as clinical risk factor, which was designed to predict operative mortality and perioperative morbidity and had been validated for patients undergoing heart surgery. As the vast majority of patients included in this series did not undergo AVR, the use of EuroSCORE-I might be controversial. Because the study cohort was enrolled since 2010, we did not use the improved EuroSCORE-II calculator [28] which was updated at 2012. Nevertheless, the EuroSCORE-I score performed reasonably well in predicting longer-term mortality in our study and continues to be the most commonly used risk score in patients with AS, especially in the absence of a superior alternative [16, 19]. Thus, our findings should be confirmed using serial change in NLR in a future prospective study.

## Conclusions

Our results demonstrate the potential utility of NLR as an inflammatory biomarker to improve risk stratification of patients with severe calcific AS. Further studies are needed to evaluate how such biomarker panels influence patient management and treatment decisions.

## Supporting Information

S1 Fig The empirical joint and marginal distributions of lymphocyte and neutrophil depicted by a scatter plot and histograms. (TIF)

S2 Fig The empirical distributions of the continuous type of variables present in Table 4 utilizing box-plots. (TIF)

S3 Fig The empirical distributions of neutrophil to lymphocyte ratio (NLR) according to sub-patient groups classified by the status of aortic valve replacement (AVR; with or without AVR) and by the status of major cardiovascular event (MACE, with or without MACE). (TIF)

S1 Table Multivariable Cox proportional hazards regression analysis regarding major cardiovascular event (MACE*) extended to include heart failure admission (all-cause mortality, non-fatal myocardial infarction and heart failure admission). (DOCX)

## Abbreviations

AS: aortic stenosis
NLR: neutrophil-to-lymphocyte ratio
EuroSCORE-I: European System for Cardiac Operative Risk Evaluation score
NT-proBNP: N-terminal pro-BNP
hs-CRP: high sensitivity C-reactive protein
MACE: major adverse cardiovascular event

## References

[1] Otto CM, Prendergast B. Aortic-valve stenosis—from patients at risk to severe valve obstruction. N Engl J Med. 2014; 371: 744–756. 10.1056/NEJMra1313875 25140960

[2] Lindman BR, Bonow RO, Otto CM. Current management of calcific aortic stenosis. Circ Res. 2013; 113: 223–237. 10.1161/CIRCRESAHA.111.300084 23833296 PMC4013234

[3] Bergler-Klein J, Klaar U, Heger M, Rosenhek R, Mundigler G, Gabriel H, et al Natriuretic peptides predict symptom-free survival and postoperative outcome in severe aortic stenosis. Circulation. 2004; 109: 2302–2308. 10.1161/01.CIR.0000126825.50903.18 15117847

[4] Cote N, Mahmut A, Bosse Y, Couture C, Page S, Trahan S, et al Inflammation is associated with the remodeling of calcific aortic valve disease. Inflammation. 2013; 36: 573–581. 10.1007/s10753-012-9579-6 23225202

[5] Jeevanantham V, Singh N, Izuora K, D'Souza JP, Hsi DH. Correlation of high sensitivity C-reactive protein and calcific aortic valve disease. Mayo Clin Proc. 2007; 82: 171–174. 10.4065/82.2.171 17290723

[6] Novaro GM, Katz R, Aviles RJ, Gottdiener JS, Cushman M, Psaty BM, et al Clinical factors, but not C-reactive protein, predict progression of calcific aortic-valve disease: the Cardiovascular Health Study. J Am Coll Cardiol. 2007; 50: 1992–1998. 10.1016/j.jacc.2007.07.064 17996566

[7] Imai K, Okura H, Kume T, Yamada R, Miyamoto Y, Kawamoto T, et al C-Reactive protein predicts severity, progression, and prognosis of asymptomatic aortic valve stenosis. Am Heart J. 2008; 156: 713–718. 10.1016/j.ahj.2008.04.011 18926152

[8] Azab B, Zaher M, Weiserbs KF, Torbey E, Lacossiere K, Gaddam S, et al Usefulness of neutrophil to lymphocyte ratio in predicting short- and long-term mortality after non-ST-elevation myocardial infarction. Am J Cardiol. 2010; 106: 470–476. 10.1016/j.amjcard.2010.03.062 20691303

[9] Cho KI, Ann SH, Singh GB, Her AY, Shin ES. Combined Usefulness of the Platelet-to-Lymphocyte Ratio and the Neutrophil-to-Lymphocyte Ratio in Predicting the Long-Term Adverse Events in Patients Who Have Undergone Percutaneous Coronary Intervention with a Drug-Eluting Stent. PLoS One. 2015; 10: e0133934 10.1371/journal.pone.0133934 26207383 PMC4514869

[10] Wang X, Zhang G, Jiang X, Zhu H, Lu Z, Xu L. Neutrophil to lymphocyte ratio in relation to risk of all-cause mortality and cardiovascular events among patients undergoing angiography or cardiac revascularization: a meta-analysis of observational studies. Atherosclerosis. 2014; 234: 206–213. 10.1016/j.atherosclerosis.2014.03.003 24681815

[11] Park BJ, Shim JY, Lee HR, Lee JH, Jung DH, Kim HB, et al Relationship of neutrophil-lymphocyte ratio with arterial stiffness and coronary calcium score. Clin Chim Acta. 2011; 412: 925–929. 10.1016/j.cca.2011.01.021 21266168

[12] Baumgartner H, Hung J, Bermejo J, Chambers JB, Evangelista A, Griffin BP, et al Echocardiographic assessment of valve stenosis: EAE/ASE recommendations for clinical practice. J Am Soc Echocardiogr. 2009; 22: 1–23; quiz 101–102. 10.1016/j.echo.2008.11.029 19130998

[13] Wang C, Yao F, Han L, Zhu J, Xu ZY. Validation of the European system for cardiac operative risk evaluation (EuroSCORE) in Chinese heart valve surgery patients. J Heart Valve Dis. 2010; 19: 21–27. 20329486

[14] O'Gara PT, Kushner FG, Ascheim DD, Casey DE Jr., Chung MK, de Lemos JA, et al 2013 ACCF/AHA guideline for the management of ST-elevation myocardial infarction: a report of the American College of Cardiology Foundation/American Heart Association Task Force on Practice Guidelines. J Am Coll Cardiol. 2013; 61: e78–140. 10.1016/j.jacc.2012.11.019 23256914

[15] Harrell FE Jr., Califf RM, Pryor DB, Lee KL, Rosati RA. Evaluating the yield of medical tests. JAMA. 1982; 247: 2543–2546. 7069920

[16] Arangalage D, Cimadevilla C, Alkhoder S, Chiampan A, Himbert D, Brochet E, et al Agreement between the new EuroSCORE II, the Logistic EuroSCORE and the Society of Thoracic Surgeons score: implications for transcatheter aortic valve implantation. Arch Cardiovasc Dis. 2014; 107: 353–360. 10.1016/j.acvd.2014.05.002 24996564

[17] Kim SI, Cho SH, Lee JS, Moon HG, Noh WC, Youn HJ, et al Clinical relevance of lymph node ratio in breast cancer patients with one to three positive lymph nodes. Br J Cancer. 2013; 109: 1165–1171. 10.1038/bjc.2013.465 23942073 PMC3778309

[18] Schemper M, Smith TL. A note on quantifying follow-up in studies of failure time. Control Clin Trials. 1996; 17: 343–346. 8889347 10.1016/0197-2456(96)00075-x

[19] Wendt D, Al-Rashid F, Kahlert P, El-Chilali K, Demircioglu E, Neuhauser M, et al Conventional aortic valve replacement or transcatheter aortic valve implantation in patients with previous cardiac surgery. J Cardiol. 2015; 66: 292–297. 10.1016/j.jjcc.2015.04.003 25975735

[20] Heymans S, Hirsch E, Anker SD, Aukrust P, Balligand JL, Cohen-Tervaert JW, et al Inflammation as a therapeutic target in heart failure? A scientific statement from the Translational Research Committee of the Heart Failure Association of the European Society of Cardiology. Eur J Heart Fail. 2009; 11: 119–129. 10.1093/eurjhf/hfn043 19168509 PMC2639409

[21] Hamirani YS, Pandey S, Rivera JJ, Ndumele C, Budoff MJ, Blumenthal RS, et al Markers of inflammation and coronary artery calcification: a systematic review. Atherosclerosis. 2008; 201: 1–7. 10.1016/j.atherosclerosis.2008.04.045 18561934

[22] Chan KL, Dumesnil JG, Tam J, Ni A, Teo K. Effect of rosuvastatin on C-reactive protein and progression of aortic stenosis. Am Heart J. 2011; 161: 1133–1139. 10.1016/j.ahj.2011.03.016 21641360

[23] Fowler AJ, Agha RA. Neutrophil/lymphocyte ratio is related to the severity of coronary artery disease and clinical outcome in patients undergoing angiography—the growing versatility of NLR. Atherosclerosis. 2013; 228: 44–45. 10.1016/j.atherosclerosis.2013.02.008 23474126

[24] Poludasu S, Cavusoglu E, Khan W, Marmur JD. Neutrophil to lymphocyte ratio as a predictor of long-term mortality in African Americans undergoing percutaneous coronary intervention. Clin Cardiol. 2009; 32: E6–E10. 10.1002/clc.20503 20014207 PMC6653698

[25] Avci A, Elnur A, Goksel A, Serdar F, Servet I, Atilla K, et al The relationship between neutrophil/lymphocyte ratio and calcific aortic stenosis. Echocardiography. 2014; 31: 1031–1035. 10.1111/echo.12534 24528173

[26] Wiegers SE. Symptomatic low-gradient severe aortic stenosis with preserved left ventricular ejection fraction: now less of a clinical conundrum. Circulation. 2013; 128: 576–578. 10.1161/CIRCULATIONAHA.113.004193 23812183

[27] Jander N, Minners J, Holme I, Gerdts E, Boman K, Brudi P, et al Outcome of patients with low-gradient "severe" aortic stenosis and preserved ejection fraction. Circulation. 2011; 123: 887–895. 10.1161/CIRCULATIONAHA.110.983510 21321152

[28] Nashef SA, Roques F, Hammill BG, Peterson ED, Michel P, Grover FL, et al Validation of European System for Cardiac Operative Risk Evaluation (EuroSCORE) in North American cardiac surgery. Eur J Cardiothorac Surg. 2002; 22: 101–105. 12103381 10.1016/s1010-7940(02)00208-7

