# Supplementary figures and images for: Prognostic Utility of Neutrophil-to-Lymphocyte Ratio on Adverse Clinical Outcomes in Patients with Severe Calcific Aortic Stenosis
Source: PLoS One. 2016 Aug 22;11(8):e0161530. doi: 10.1371/journal.pone.0161530 (PMC4993489; doi:10.1371/journal.pone.0161530)

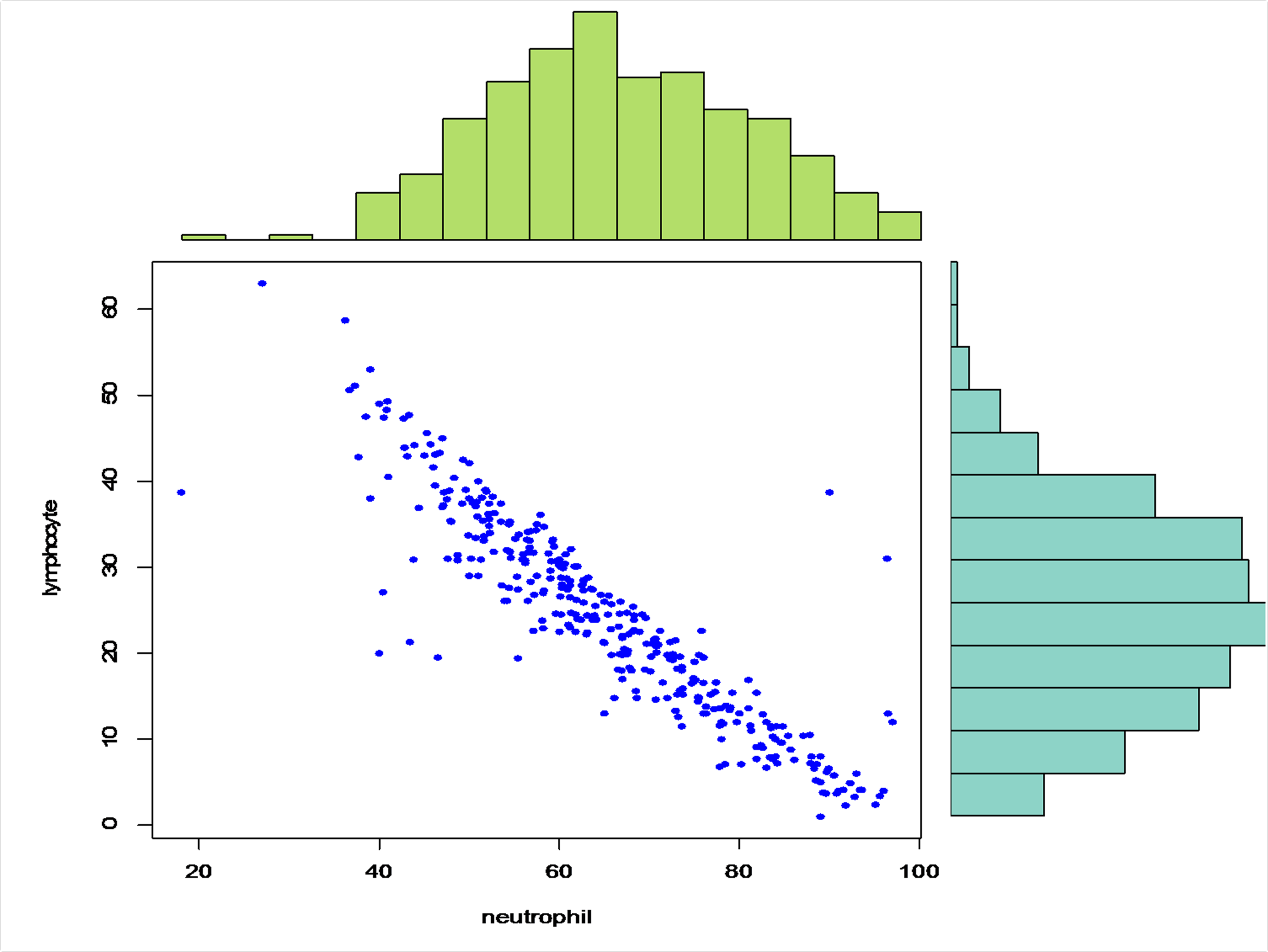

Supplement: S1 Fig — (TIF) [file pone.0161530.s001.tif]

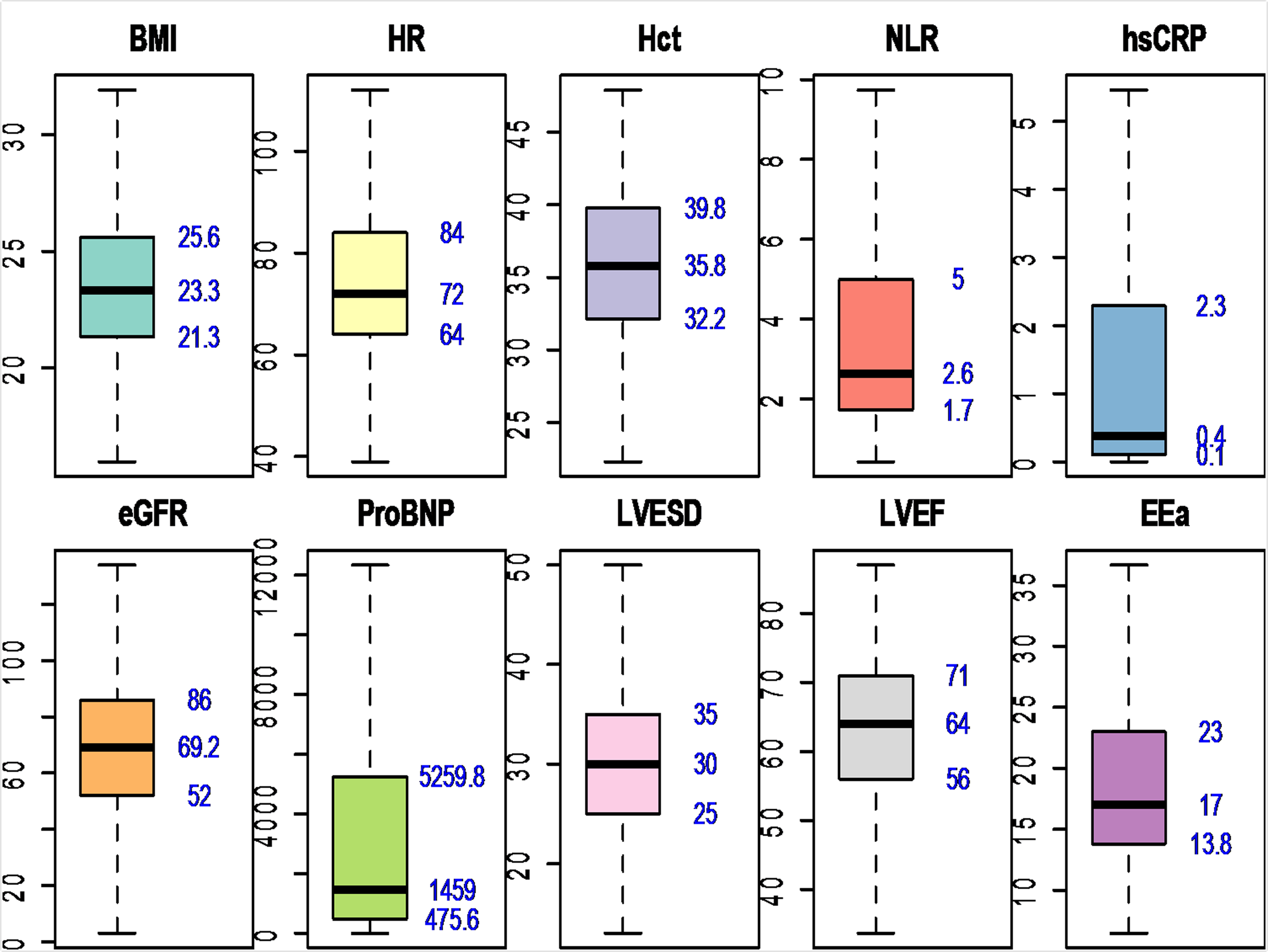

Supplement: S2 Fig — (TIF) [file pone.0161530.s002.tif]

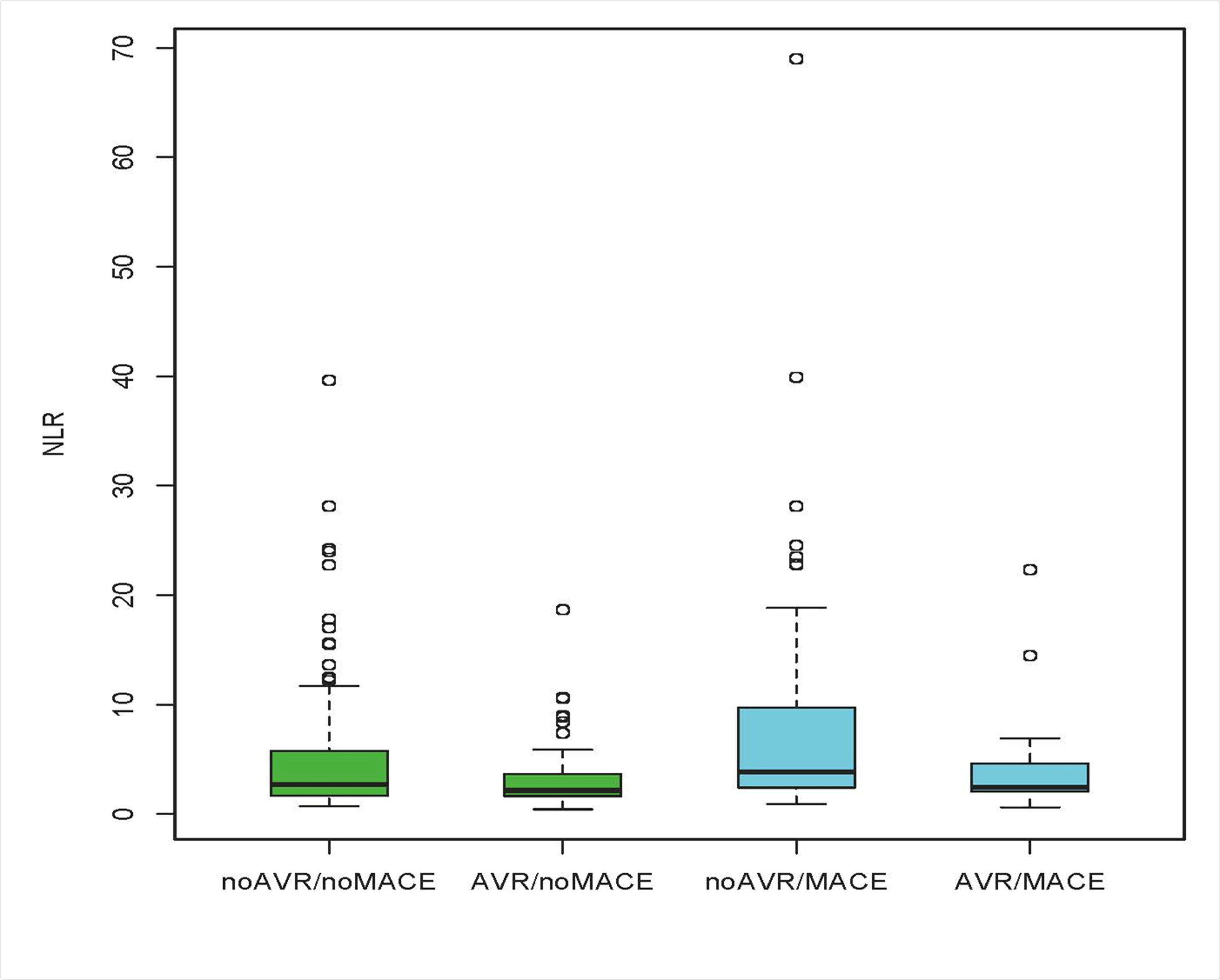

Supplement: S3 Fig — (TIF) [file pone.0161530.s003.tif]
