# Supplementary material for: Prognostic Utility of Neutrophil-to-Lymphocyte Ratio on Adverse Clinical Outcomes in Patients with Severe Calcific Aortic Stenosis
Source: PLoS One. 2016 Aug 22;11(8):e0161530. doi: 10.1371/journal.pone.0161530 (PMC4993489; doi:10.1371/journal.pone.0161530)
Supplement: S1 Table — (DOCX) [file pone.0161530.s004.docx]

**S1 Table1. Multivariable Cox proportional hazards regression analysis regarding major cardiovascular event (MACE*) extended to include heart failure admission** (all-cause mortality, non-fatal myocardial infarction and heart failure admission)

|  | Multivariable | | |
| --- | --- | --- | --- |
|  | HR | 95% CI | *p*-value |
| EuroSCORE-I | 1.04 | 1.02 to 1.06 | <0.001 |
| Aortic valve replacement, n (%) | 0.79 | 0.55 to 1.14 | 0.21 |
| Neutrophil to lymphocyte ratio | 1.04 | 1.03 to 1.06 | <0.001 |

**S1 Figure. The empirical joint and marginal distributions of lymphocyte and neutrophil depicted by a scatter plot and histograms**

**S2 Figure. The empirical distributions of the continuous type of variables present in Table 4 utilizing box-plots**

BMI, body mass index; HR, heart rate; Hct, hematochrit; NLR, neutrophil-to-lymphocyte ratio; hs-CRP, high sensitivity C-reactive protein; eGFR, estimated glomerular filtration rate; ProBNP, n-terminal pro brain natriuretic peptide; LVESD, left ventricular end-systolic diameter; LVEF, left ventricular ejection fraction; E, peak early diastolic mitral filling velocity; Ea, mitral annular velocity

**S3 Figure. The empirical distributions of neutrophil-to-lymphocyte ratio (NLR) according to sub-patient groups classified by the status of aortic valve replacement (AVR; with or without AVR) and by the status of major adverse cardiovascular event (MACE; with or without MACE)**
